# BMC Biophysics reviewer acknowledgement 2014

**DOI:** 10.1186/s13628-015-0017-7

**Published:** 2015-02-10

**Authors:** Catherine J Potenski

**Affiliations:** BioMed Central, 233 Spring Street, New York, 10013-1578, NY USA

## Abstract

The editors of *BMC Biophysics* would like to thank all our reviewers who have contributed to the journal in Volume 7 (2014).

**Nathan Baker**

USA

**Andrew Burnett**

UK

**Miguel Castanho**

Portugal

**Martin Depken**

Netherlands

**Markus Dittrich**

USA

**Pemra Doruker**

Turkey

**Robert Falconer**

UK

**Dmitri Fedorov**

Japan

**Michael Feig**

USA

**Susan M Gasser**

Switzerland

**Gernot Guigas**

Germany

**Gary Huber**

USA

**Themis Lazaridis**

USA

**Timothy Lezon**

USA

**Ray Luo**

USA

**Christopher Mathews**

USA

**Andrew J Mccammon**

USA

**Ralf Metzler**

Germany

**Christine Milcarek**

USA

**James Milner-White**

UK

**Dariush Mohammadyani**

USA

**Subhodeep Moitra**

USA

**Paul Moss**

UK

**Edward Parrott**

UK

**Sanbo Qin**

USA

**Arvind Ramanathan**

USA

**Markus Sauer**

Germany

**Michael Schlierf**

Germany

**Sundar Raman Subramanian**

USA

**Attila Szabo**

USA

**Vladimir Turzhitsky**

USA

**Katarzyna Tych**

UK

**Maximilian Ulbrich**

Germany

**Hongyun Wang**

USA

**Wei Yang**

USA

